# The relationship between daytime napping and glycemic control in people with type 2 diabetes

**DOI:** 10.3389/fendo.2024.1361906

**Published:** 2024-03-05

**Authors:** Jinjin Yuan, Jinle Wang, Yingdan Chen, Min Zhang, Aimei Zhao, Jing Du, Jiahui Zhang, Fan Liu, Yueying Wang, Pei Chen, Bingqian Zhu

**Affiliations:** ^1^ School of Nursing, Shanghai Jiao Tong University, Shanghai, China; ^2^ Department of Nursing, Lujiazui Community Health Service Center, Shanghai, China; ^3^ Department of Nursing, Beixinjing Community Health Service Center, Shanghai, China; ^4^ Department of Nursing, The Second People’s Hospital of Kashgar Region, Xinjiang, China; ^5^ Department of Psychiatry, Tongji University Affiliated Tongji Hospital, Shanghai, China; ^6^ College of Nursing, University of Illinois Chicago, Chicago, IL, United States

**Keywords:** diabetes, nap, sleep, glycemic control, HbA1c

## Abstract

**Aim:**

To examine the association between napping characteristics and glycemic control in people with type 2 diabetes.

**Design:**

This study used a cross-sectional design.

**Methods:**

A convenience sample of people with type 2 diabetes (N=226) were included. Glycemic control was indicated by HbA1c which was measured by A1C Now®+. Napping characteristics including napping frequency, duration, timing, and type were measured by validated questionnaires. Other variables, such as insomnia, cognitive impairment, and depression were measured by the Insomnia Severity Index, Montreal Cognitive Assessment, and Patient Health Questionnaire-9, respectively. Multivariate linear regression analyses were performed.

**Results:**

The sample consisted of 122 women (54.0%), with a median age of 67 years. Their median HbA1c was 6.8%. No significant relationship was found between napping frequency and HbA1c. Among nappers, after controlling for covariates, long napping duration (≥60 min) and morning napping were both associated with poorer glycemic control. Compared with appetitive napping, restorative napping was associated with better glycemic control.

**Conclusion:**

Daytime napping (e.g., duration and type) is an important modifiable factor for glycemic control in people with type 2 diabetes. This study provides new insights into the relationship between napping and glucose management among people with diabetes.

## 1 Introduction

In 2021, one in ten adults was living with diabetes, and around 90%-95% were diagnosed with type 2 diabetes (T2D) (1). Diabetes was among the top 10 causes of death (2), causing significant burdens to the individuals and society. Adequate glycemic control is the key to preventing or delaying the development of diabetic complications (3). Hemoglobin A1c (HbA1c), which reflects the overall glycemic control over the past two to three months, has been used as the primary indicator for diabetes management (4). Traditional factors, including demographics (e.g., age and sex) (5), clinical characteristics (e.g., BMI, diabetes duration, and treatment regimen) (5–7), lifestyle factors (e.g., smoking and drinking) (8), and psychosocial factors (e.g., depression and cognitive function) (9, 10) have been associated with HbA1c. Emerging evidence indicates that sleep may also play a role in the development and management of diabetes (11, 12).Sleep is a complex and dynamic process during which there is a reduced responsiveness to external stimulation. In most cases (except for shift workers), it includes nighttime sleep and daytime napping. In recent years, researchers started to examine the impact of napping on human health. A nap is defined as any sleep period with a duration of less than 50% of the average major sleep period. In addition to the traditionally assessed dimensions (e.g., frequency, duration), type and timing of napping are also important dimensions. The type of napping can be categorized as replacement (i.e., a nap taken in response to sleep loss), prophylactic napping (i.e., a nap taken in anticipation of sleep loss), and appetitive napping (i.e., nap for enjoyment or habitual napping such as siesta) (13). The category of “replacement” and “prophylactic” napping may be confusing for participants frequently experiencing sleep deprivation. Thus, they have been labeled as “restorative” (14, 15). Among people with T2D, disturbed nighttime sleep (e.g., short sleep duration, poor sleep quality, and insomnia symptoms) have been associated with poorer glycemic control (12, 16). Napping, which is very common in many cultures (e.g., China), may also play an important role in cardiometabolic health (17).

Several studies have examined the relationship between napping and glycemic control in people with T2D (18–21), with inconsistent results. Specifically, Bawadi, et al. found that those who “sometimes, frequently, or always” napped had an increased risk of poor glycemic control than those who “never or rarely” napped (20). This finding is similar to the one found in another study, showing an approximately threefold risk of having poor glycemic control in nappers compared with non-nappers (21). In contrast, Gozashti, et al. reported that those who napped had better glycemic control than those who did not nap (18). In another study, taking midday naps in short sleepers (sleep duration<5h) was associated with a reduced risk for poor glycemic control (19). Although previous studies conducted in people with T2D provided evidence about the association between napping and glycemic control, they have been mostly focused on one single dimension of napping (i.e., frequency), neglecting the complexity of napping. Studies conducted in other populations found that longer napping duration was associated with hyperglycemia in pregnant women (22) and increased risk for impaired fasting glucose and diabetes in retired workers (23). Based on the above evidence, there is a need to provide a complete picture of the association between napping and glycemic control among people with T2D. This study aims to: 1) describe the napping characteristics of people with T2D and 2) examine the relationship between napping with glycemic control. Findings from this study may provide more evidence for diabetes management and add to current knowledge about the relationship between sleep and diabetes.

## 2 Materials and methods

### 2.1 Study design

A cross-sectional design was used. The study was approved by the Institutional Review Board of Shanghai Jiao Tong University School of Medicine (#SJUPN-201811). All participants signed a paper informed consent form before completing the questionnaires.

### 2.2 Sample size calculation

Sample size calculation was performed by G*Power 3.1 (Franz Faul, Germany). α (two-tail) and 1-β was set at 0.05 and 0.8, respectively. A total of 15 predictors were considered when calculating *a priori* sample size, including four napping variables, two night sleep variables (i.e., insomnia and sleep duration) (12, 16), and nine traditional factors as indicated in the earlier section. Based on a study conducted in people with type 1 diabetes examining the association between napping frequency and HbA1c level, the effect size was 0.74 (95%CI=0.09-1.40) (24). In this study, the lower limit of 0.09 was used to ensure adequate power. We assumed that each napping variable would be associated with HbA1c, with a small effect size. F test was used for the calculation, which resulted in a minimum sample size of 222. A total of 226 participants were included in the final analysis.

### 2.3 Participants

Participants were recruited using a convenience sampling method. The inclusion criteria were: (a) 18 years of age or older; (b) being diagnosed as T2D according to the WHO diagnosis criteria: random plasma glucose concentration ≥11.1mmol/L, and/or fasting plasma glucose concentration ≥7.0mmol/L, and/or 2-hour postprandial plasma glucose concentration ≥11.1mmol/L, accompanied by diabetic-related symptoms (25), or self-reported T2D (as determined by current use of antidiabetic drugs or insulin), or confirmed by a physician; (c) did not change the therapeutic regimen during the past three months.

The exclusion criteria were self-reported: (a) having serious chronic diseases (e.g., severe cardio-cerebrovascular disease and chronic kidney disease); (b) having severe diabetic complications (e.g., severe retinopathy and nephropathy), (c) pregnant women; (d) on night shift; (e) travelled crossed the time zone in the past week; (f) having mental illness (e.g., schizophrenia) or severe cognitive impairment, causing difficulties in obtaining informed consent.

### 2.4 Procedures

**Figure 1:**
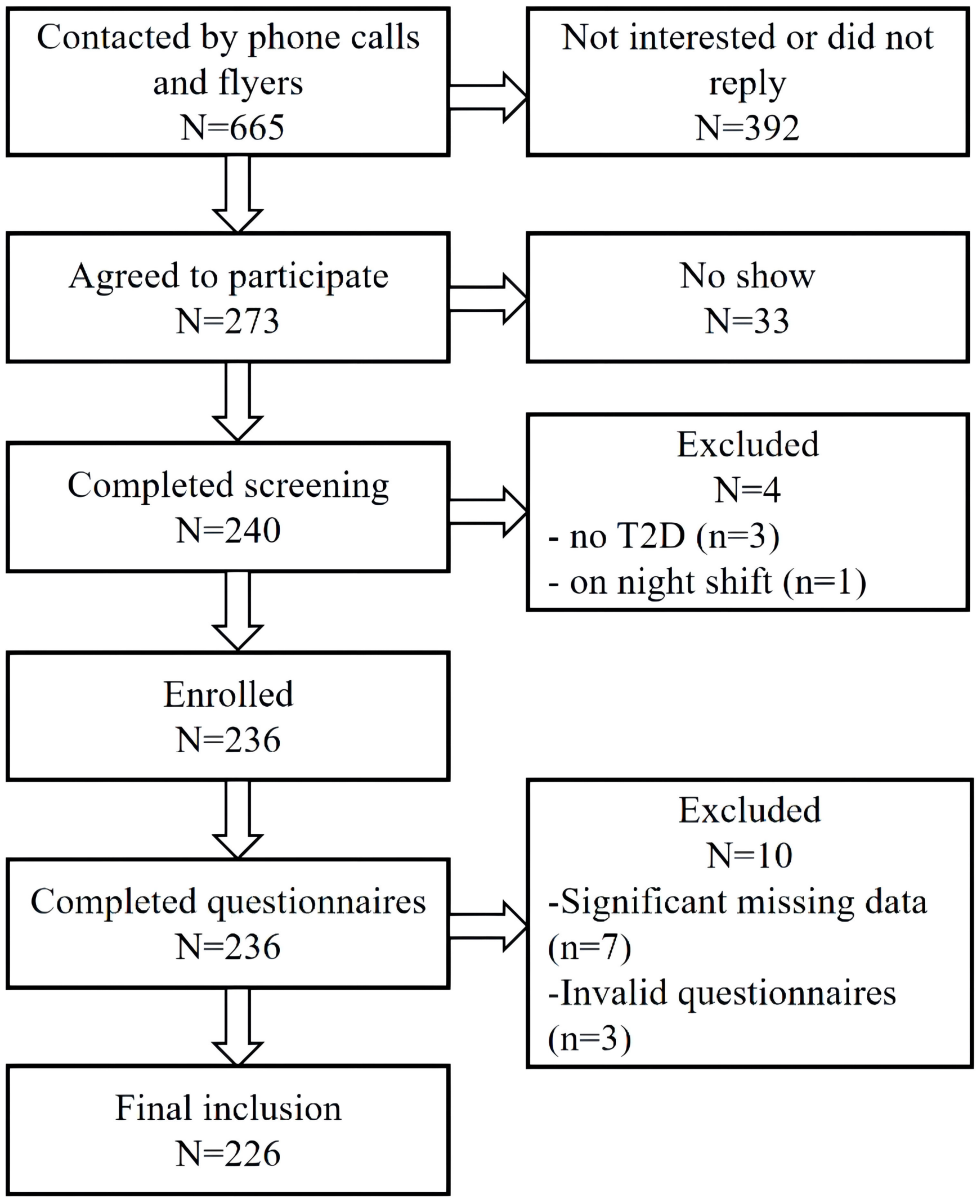
Participant recruitment flowchart.

Participants were recruited from two community healthcare centers in Shanghai and one in Henan, China from May 2023 to July 2023. The process of recruitment is shown in **Figure 1**. Briefly, 665 participants were contacted, with 273 agreed to participate. Among them, 240 completed the screening, and 236 were enrolled. Ten were excluded due to significant missing data on key questionnaires or invalid responses (response with a clear pattern).

Data were collected using paper-and-pencil questionnaires. The participants were instructed to fill out the questionnaires in a quiet room. Two graduate students were present to answer questions the participants had. The following questionnaires were used to measure variables of interest, including demographic, clinical, psychosocial, and lifestyle characteristics, sleep (e.g., overall sleep health and insomnia) and napping behaviors (e.g., napping frequency, duration, timing and type).

### 2.5 Measurement

#### 2.5.1 Glycemic control

Glycemic control was assessed by HbA1c and poor glycemic control was defined as HbA1c≥7%, consistent with previous evidence (20). HbA1c was measured by A1C Now®+ (PTS Diagnostics) using a fingertip blood sample (26).

#### 2.5.2 Napping

Items from the Napping Behavior Questionnaire were used to assess napping (27) frequency and duration. Napping frequency was measured by “On average how many days do you take daytime nap during a typical week”? The response included (a) <1, (b) 1-2, (c) 3-4, (d) 5-6, and (e) >6. It was categorized into (a) <1, (b) 1-2, (c) 3-4, (d) 5-7 during data analysis based on previous evidence (28). Napping duration was measured by “How long is your typical nap”? The response included (a) <15min, (b) 15-30min, (c) 30-45min, (d) 45-60min, and (e) >60min. It was dichotomized as long napping duration (a) yes (≥60 min) and (b) no (<60 min) based on previous evidence (29).

The timing of napping was measured by “What time did your nap usually occur”? The response included (a) in the morning (before 12 pm), (b) in the afternoon (12 pm to 4:30 pm), (c) in the evening (4:30 pm to 9 pm) (30). The type of napping was measured by “Why do you usually nap”? The response included (a) “purely due to habit/enjoyment/convenience, and not related to lack of sleep at night” (appetitive), (b) “didn’t get enough sleep or didn’t sleep well the night before” (replacement), and (c) “expecting lacking sleep at night” (prophylactic) (31). Consistent with previous studies, (b) and (c) were combined and labeled as “restorative” napping (14, 15).

#### 2.5.3 Other variables

##### 2.5.3.1 Sleep-related covariates

###### 2.5.3.1.1 Insomnia

Insomnia was measured by the Insomnia Severity Index (ISI). The ISI contains 7 items assessing the severity and consequences of insomnia in the past two weeks. Each item is scored on a 5-point Likert scale (0 to 4). Adding all items results in the total score, ranging from 0 to 28. Higher total scores indicate severer insomnia symptoms (32). The ISI has good internal consistency (Cronbach’s α=0.90) and a score of 10 or over has been used as the cut-off for clinically significant insomnia (33). The Chinese version of ISI showed good internal consistency (Cronbach’s α=0.84) (34). In this sample, the internal consistency of ISI was 0.92.

###### 2.5.3.1.2 Sleep duration

Sleep duration was assessed by asking the participants “In the past week, how many hours of sleep did you usually get on weekdays and weekends”? Sleep duration was calculated as (5*sleep duration on weekday+2*sleep duration on weekend)/7 (h). Sleep duration < 6h was considered short sleep (35).

##### 2.5.3.2 Cognitive function

Cognitive function was assessed by the Montreal Cognitive Assessment Beijing Version (MoCA). It measures eight domains of cognitive function. Adding scores for each domain results in a total score of 0-30. One point was added to the total score if the participants had an education years ≤ 12. A total score ≤ 25 indicates mild cognitive impairment (36). The MoCA Beijing version showed good sensitivity and validity in screening cognitive impairment in elderly adults (37). In this sample, the internal consistency 0.65.

##### 2.5.3.3 Depression

The Patient Health Questionnaire-9 (PHQ-9) was used to assess depressive symptoms during the past two weeks. The PHQ-9 consists of nine items, with each scored on a 4-point Likert scale ranging from 0 (not at all) to 3 (nearly every day) (38). Adding all items results in a total score of 0 to 27. Higher scores indicate severer depressive symptoms. A score of 5 or over has been used as the cut-off for depression. The Chinese version of PHQ-9 has been demonstrated good internal consistency (Cronbach’s α=0.89). In this sample, the internal consistency 0.78.

##### 2.5.3.4 Demographic and clinical characteristics

A questionnaire was developed by the research team to measure demographic and clinical characteristics of the participants, such as age, sex, education level, marital status, employment status, smoking, and drinking. We also measured height and weight using objective scales. Clinical characteristics included self-report diagnosis of hypertension, family history of diabetes, diabetes duration, and treatment regimen for diabetes.

### 2.6 Data analysis

The SPSS 24.0 (IBM, Armonk, NY, USA) and OriginPro 9.9 (OriginLab Corp., Northampton, MA, USA) were used for data analysis and visualization. Missing data, normal distribution, and outliers were checked prior to data analysis. Mean substitution was used when missing data was less than 5%. Continuous variables were presented as mean (SD) if normally distributed and as median and interquartile range (IQR) otherwise. Categorical variables were presented as frequency (%). Independent sample t-test or Mann-Whitney U test, analysis of variance (ANOVA) or Kruskal-Wallis H test, and Chi-square test were used for group comparison. Spearman correlation analysis was performed to examine the association between HbA1c and other continuous variables. Multivariate linear regression analysis was performed to examine the association between napping and HbA1c while controlling for *a priori* covariates including age, sex, BMI, treatment regimen, diabetes duration (5, 39), cognitive impairment, depression (9, 10), and night sleep (e.g., sleep duration and insomnia symptoms) (12, 16). Variables associated with HbA1c at p<0.2 in the bivariate analyses (i.e., hypertension) were also controlled. Statistical significance was set at p<0.05 (two-tailed).

## 3 Results

### 3.1 Characteristics of participants

The median age of the participants was 67 years, and 122 (54.0%) were females. The majority of the participants were retired (88.1%). The mean BMI was 24.7 (SD 3.5) kg/m^2^. About a half of the participants had a family history of diabetes (46.5%), and oral medication was the most commonly used treatment regimen (68.1%). Participants had a median diabetes duration and HbA1c of 10 years and 6.8%, respectively. Detailed characteristics of the participants are shown in **Table 1**.

**Table 1:** Characteristics of participants (N=226).

Variables	Mean (SD) or Median (IQR) or N (%)
Age (years)	67 (61-75)
Sex (female)	122 (54.0%)
Education Junior high or below High school level University level	112 (49.6%) 92 (40.7%) 22 (9.7%)
Marital status Married Unmarried/divorced/widowed	184 (81.4%) 42 (18.6%)
Work status Currently working Retired/unemployed	27 (11.9%) 199 (88.1%)
BMI (kg/m^2^)	24.7 (3.5)
Current smoker (yes)	40 (17.7%)
Current drinker (yes)	41 (18.1%)
Hypertension (yes)	137 (60.6%)
Family history of diabetes (yes)	105 (46.5%)
Diabetes duration (years)	10.0 (5.0-18.0)
Therapeutic regimen Lifestyle intervention Oral medication(s) Insulin Oral medication plus insulin	24 (10.6%) 154 (68.1%) 11 (4.9%) 37 (16.4%)
MoCA Mild cognitive impairment (MoCA ≤ 25) (yes)	23 (20, 26) 159 (70.4%)
HbA1c (%) Poor glycemic control (HbA1c ≥7%) (yes)	6.8 (6.1-7.9) 100 (44.2%)

BMI, body mass index; IQR, interquartile range; SHI, Sleep Health Index; SD, standard deviation; ISI, Insomnia Severity Index; MoCA, Montreal Cognitive Assessment; PHQ-9, Patient Health Questionnaire-9.

### 3.2 Napping and sleep-related characteristics of the participants

In this sample (N=226), 20.4% (n=46) were non-nappers. Among nappers (n=180), 33.9% had long napping duration. A majority had afternoon napping (90.0%). The common type of napping was appetitive napping (72.8%). In addition, the median sleep duration was 6.3 hours, with 32.3% having short sleep duration (<6h). The median score of ISI was 3.0 (IQR 1.0-8.0). Detailed characteristics are shown in **Table 2**.

**Table 2:** Napping and sleep-related characteristics of the participants.

Variables	Mean (SD) or Median (IQR) or N (%)
Napping frequency None 1-2 days/week 3-4 days/week 5-7 days/week	46 (20.4%) 38 (16.8%) 33 (14.6%) 109 (48.2%)
Long napping duration **^a^** Yes No	61 (33.9%) 119 (66.1%)
Timing of napping **^a^** morning (before 12 pm) afternoon (12 pm to 4:30 pm) evening (4:30 pm to 9 pm)	15 (8.3%) 162 (90.0%) 3 (1.7%)
Type of napping **^a^** Appetitive napping Restorative napping	131 (72.8%) 49 (27.2%)
Sleep duration (h) Short sleep duration (yes)	6.3 (5.5-7.5) 73 (32.3%)
ISI Insomnia (yes)	3.0 (1.0-8.0) 37 (16.4%)

^a^N=180; IQR, interquartile range; SD, standard deviation; ISI, Insomnia Severity Index.

### 3.3 Bivariate association between participant characteristics (categorical variables) and HbA1c

Bivariate analyses showed no significant difference in HbA1c between different sex, education, marital status, work status, smoking, drinking, hypertension, family history of diabetes, and insomnia (**Table 3**). In comparison, significant differences in HbA1c were detected between people using different therapeutic regimen (p=0.022) and those with or without mild cognitive impairment (p=0.021) (**Table 3**).

**Table 3:** Bivariate analysis examining relationship between categorical variables (not including napping characteristics) and HbA1c level (N=226).

Categorical variables	HbA1c (%)	p
	median (IQR)	
Sex Female Male	6.8 (6.0-7.8) 6.9 (6.2-8.1)	0.328
Education Junior high or below High school level University level	6.9 (6.2-8.2) 6.5 (6.0-7.8) 6.9 (6.5-7.7)	0.370
Marital status Married Unmarried/divorced/widowed	6.8 (6.1-7.9) 6.8 (6.2-7.9)	0.940
Work status Currently working Retired/unemployed	6.8 (6.2-8.2) 6.8 (6.1-7.8)	0.659
Current smoker Yes No	6.8 (6.2-8.0) 6.8 (6.1-7.8)	0.745
Current drinker Yes No	7.0 (6.2-8.2) 6.8 (6.1-7.8)	0.475
Hypertension Yes No	6.7 (6.0-7.8) 6.9 (6.3-7.9)	0.165
Therapeutic regimen Lifestyle intervention Oral medication Insulin Oral medication plus insulin	6.4 (6.0-7.4) 6.7 (6.0-7.8) 7.4 (6.7-8.8) 7.3 (6.5-8.4)	0.022
Family history of diabetes Yes No	6.8 (6.2-7.9) 6.8 (6.0-8.0)	0.714
Short sleep duration Yes No	6.7 (6.1-8.3) 6.9 (6.1-7.8)	0.739
Insomnia Yes No	6.9 (5.7-8.5) 6.8 (6.2-7.8)	0.742
Mild cognitive impairment Yes No	7.0 (6.2-8.0) 6.5 (5.9-7.2)	0.021
Depression Yes No	7.0 (6.2-8.6) 6.7 (6.1-7.6)	0.102

MoCA, Montreal Cognitive Assessment; PHQ-9, Patient Health Questionnaire-9.

**Figure 2:**
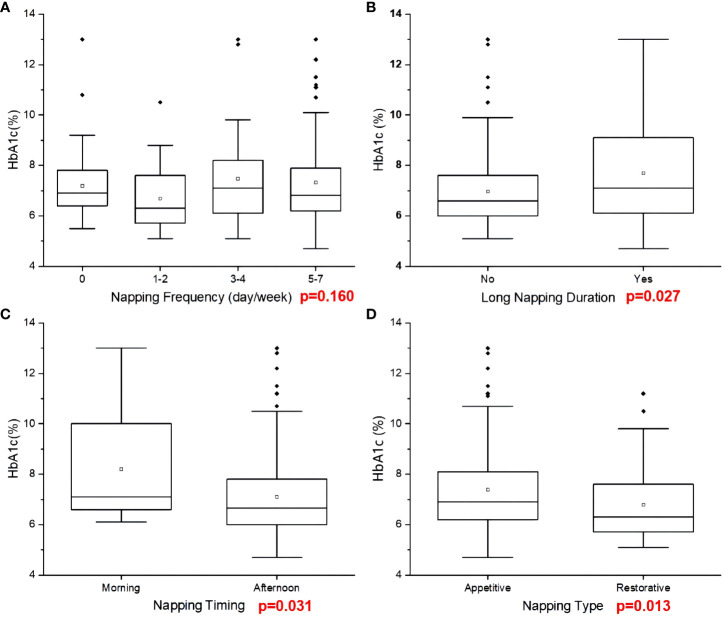
HbA1c level based on napping characteristics.

HbA1c level in participants with different napping frequency was not significantly different (p>0.05) (**Figure 2**). However, participants with long napping duration (≥60min/day) had a higher level of HbA1c (p=0.027). Compared with morning napping, afternoon napping was related to lower HbA1c (p=0.031). Those with restorative napping had significantly lower HbA1c level than those with appetitive napping (p=0.013) (**Figure 2**). Detailed data are shown in **Supplementary Table 1**.

### 3.4 Bivariate association between participant characteristics (continuous variables) with HbA1c level

Spearman correlation analyses showed that diabetes duration and MoCA total scores were significantly corelated to HbA1c level (p<0.001 and p=0.002) (**Table 4**).

**Table 4:** Correlation between participant characteristics (continuous variables) with HbA1c level (N=226).

Variables	r	p
Age (year)	0.091	0.174
BMI (kg/m^2^)	-0.039	0.561
Diabetes duration (year)	0.250	<0.001
Sleep duration (h)	0.052	0.434
ISI	-0.033	0.627
MoCA	-0.201	0.002
PHQ-9	0.090	0.177

BMI, body mass index; ISI, Insomnia Severity Index; MoCA, Montreal Cognitive Assessment; PHQ-9, Patient Health Questionnaire-9.

### 3.5 Multivariate association between napping characteristics and HbA1c level

**Table 5** shows the multivariate linear regression results of predictors of HbA1c. Collectively, the independent variables accounted for 22.4% of the variance in HbA1c level (R^2 =^ 22.4%, p=0.001). Controlling for covariates, long napping duration (≥60min/day) was positively associated with HbA1c level (B=0.640, 95%CI=0.129, 1.151, p=0.014). Morning napping (vs. afternoon napping) was positively associated with HbA1c level (B=0.992, 95%CI=0.112, 1.872, p=0.027). While restorative napping (vs. appetitive) was associated with lower HbA1c level (B=-0.670, 95%CI=-1.269, -0.072, p=0.028).

**Table 5:** Multivariate linear regression model of predictors of HbA1c level among nappers ^a^.

Variables	B	β	95% CI	p
Age (years)	-0.026	-0.157	-0.053, 0.002	0.070
Sex (female)	0.004	0.001	-0.512, 0.519	0.989
BMI (kg/m^2^)	-0.044	-0.086	-0.121, 0.034	0.265
Hypertension (yes)	0.317	0.091	-0.216, 0.849	0.242
Therapeutic regimen (Ref: Lifestyle intervention) Oral medication Insulin Oral medication plus insulin	-0.359 0.081 -0.098	-0.099 0.011 -0.021	-1.153, 0.434 -1.200, 1.362 -1.137, 0.940	0.373 0.901 0.852
Diabetes duration (year)	0.055	0.277	0.020, 0.091	0.003
Mild cognitive impairment (yes)	0.291	0.078	-0.270, 0.853	0.307
Depression (yes)	0.243	0.069	-0.343, 0.830	0.414
Sleep duration (h)	0.078	0.067	-0.109, 0.266	0.410
Insomnia (yes)	0.197	0.042	-0.634, 1.027	0.641
Napping frequency ^a^ (Ref: 5-7 days/week) 1-2 days/week 3-4 days/week	-0.368 0.484	-0.089 0.108	-1.015, 0.278 -.195, 1.164	0.262 0.161
Long napping duration ^a^ (yes)	0.640	0.179	0.129, 1.151	0.014
Napping timing ^a^ (morning, before 12 pm)	0.992	0.163	0.112, 1.872	0.027
Napping type ^a^ (restorative)	-0.670	-0.176	-1.269, -0.072	0.028

a N=177, three participants with evening napping were excluded from the analysis; B, unstandardized coefficient; β, standardized coefficient; CI, confidence interval.

## 4 Discussion

In this study, we first described the napping characteristics of people with T2D and then examined the relationship between napping with glycemic control. We found that long napping duration (≥60 min) and morning napping were both associated with poorer glycemic control as indicated by higher HbA1c. In addition, restorative napping, compared with appetitive napping, was associated with better glycemic control. Findings from this study may provide more evidence for diabetes management and add to current knowledge about the relationship between sleep and diabetes.

In this sample, 20.4% were non-nappers, comparable to T2D patients in Qatar (19.4%, aged 52 years, 60% females) (20) but significantly lower than that in a Japanese T2D sample (54.5%, aged around 66 years) and Ethiopian T2D sample (68.8%, over 60% aged 45 years and older, 46% females) (19, 40). Among nappers, half of them (48.2%) had a high napping frequency of 5-7 days/week. Similarly, in the Japanese T2D sample, 15.4% napped 4-6 days/week, and 67.4% napped daily (19). In comparison, in the Ethiopian sample, around 39% reported frequently/always napped (40). The inconsistency may be due to different categorization of napping frequency, cultural differences in napping, and other sociodemographic factors. For example, napping was considered a behavior good for health in the elderly in China (41). In addition, around 90% of our participants were retired/unemployed as compared to 11% in the Ethiopian sample. People who were retired or unemployed may have more opportunities to take naps. Around 34% of our sample had long napping duration (≥60 min), in line with a previous study conducted in a Chinese T2D sample (31.1%) (42). The majority of our sample napped in the afternoon (90.0%) and were appetitive nappers (72.8%). Few studies have investigated the timing and type of napping in people with T2D. Among middle-aged adults in Switzerland (aged 61 years, 58% females), 42.5% took naps in the afternoon (12 pm-4:30 pm), 23.3% in the morning, and 31.3% in the evening (30). Climate difference between the two countries might explain the inconsistent finding as sleep propensity and pattern were related to body temperature (43) which could be affected by climates (44). A study in the U.S. showed that college students tended to be restorative nappers rather than appetitive nappers (78% vs. 22%) (45), in contrast to our finding. Participants in this study were middle-aged or older adults and were mainly retired (88.1%). They were less likely to have nighttime sleep loss and thus had lower need for restorative napping. In comparison, they might have more free time to nap for enjoyment and easier to form a habit (46).

In this sample, we did not find a significant association between napping frequency with HbA1c. Association between napping frequency and glycemic metabolism has been examined in a variety of populations, demonstrating conflicting results. One study conducted in pregnant women (aged 23 years) found that napping frequency did not have a significant association with 1-h OGTT values or hyperglycemia (N=63) (47). High napping frequency was found associated with an increased risk of developing T2D (N=435,342) (aged 56 years, 55% females) (48). Compared with non-nappers, people with regular napping were 1.3 times more likely to have poor glycemic control (N=12,997) (aged 59 years, 38% females) (49). Based on a recent meta-analysis, compared with non-nappers, nappers had a 20% increased risks of developing T2D (50). How napping frequency affect glucose metabolism has not been clear. Alterations of melatonin may be a potential mechanism. Melatonin level was found to be elevated temporarily after a 2-hour nap during 12-hour simulated night work (51). As melatonin could improve insulin resistance, napping may thus be beneficial for glycemic control (52). Adiposity may play another role as it attenuated the association between napping and diabetes risk (53). In this study, we controlled for BMI and did not find a significant relationship. This null finding may be explained by the relatively small sample. Future studies with a larger sample are needed.

In this study, we found that long napping duration was associated with higher HbA1c. Long napping duration may be a reflection of impaired nighttime sleep. We thus controlled for the potential confounding effect of night sleep (e.g., sleep duration and insomnia symptoms). The association remained significant. This finding is consistent with the one from a study (aged 64 years, 53% females) indicating that those having napping duration of over 1 hour had a higher risk of developing diabetes and having impaired fasting glucose (23). Several reasons may explain this finding. Based on a meta-analysis, napping duration of over 1 hour was associated with an increased occurrence of obesity, possibly caused by decreased energy expenditure (54), and obesity was a predictor of poor glycemic control (55). In addition, long daytime napping may influence nighttime sleep by disturbing circadian rhythmicity (56). For example, long napping duration was found related to later bedtimes (57), increased nighttime awakenings, shorter nighttime sleep, lower sleep efficiency (58), and poorer sleep quality (59). Impaired night sleep may affect glucose metabolism through increased nocturnal cortisol concentration and sympathetic nervous system activity (60).

Compared with afternoon napping, morning napping was associated with a higher level of HbA1c. Current evidence on the timing of napping in people with T2D has been limited. Typically, napping is most likely to occur in the afternoon accompanied with the highest level of daytime sleepiness or sleep propensity (61). Afternoon napping thus may be of better quality and have bigger restorative effect that meets people’s needs of relieving sleepiness. In comparison, morning napping may be indicative of disturbed night sleep. Overall, the detrimental effect of impaired nocturnal sleep may outweigh the restorative effect of daytime napping. However, our analysis controlled for potential confounding effect of nocturnal sleep, suggesting that the effect of napping could be independent of night sleep. How the timing of napping may affect cardiometabolic health remains unclear, warranting further investigation.

To the best of our knowledge, this study was among the first that examined the relationship between type of napping and glucose metabolism. Compared with appetitive napping, restorative napping was related to better glycemic control. Restorative naps are naps taken in compensation for previous sleep loss, while appetitive naps indicate naps taken purely for psychological satisfaction or enjoyment other than sleep needs. Based on a previous study, appetitive nappers had more stage 1 sleep than restorative nappers (62), suggesting worse sleep quality. In addition, restorative nappers tend to take naps on those days when for the preceding one or more days they reported lacking sleep (45). Sleep deprivation may induce inflammation and cytokine secretion (e.g., IL-1β and TNF-α) in diabetic islets, impairing β cell function and insulin secretion (63, 64). As daytime napping is associated with immune recovery by preserving energy (65), taking restorative naps in response to “sleep debt” may return immune cytokines to baseline levels accompanied with subsequent nighttime sleep (62). Interestingly, based on previous evidence, appetitive nappers had higher sleep needs, slept longer than restorative nappers at night. They nap frequently without shortened nighttime sleep, but can also fall asleep easily (62). Indeed, the nighttime sleep duration of appetitive nappers in this study was longer than that in restorative nappers (6.6 h vs. 5.8 h), and appetitive nappers had a higher napping frequency than that in restorative nappers (69.5% vs. 36.7% napped 5-7 day/weeks). It is likely that in appetitive nappers, their longer sleep time throughout the day could decrease energy expenditure (54) and disturb circadian rhythmicity (56) and thus contribute to poorer glycemic control.

A major strength of this study was a thorough investigation of napping, especially the timing and type of napping, among patients with T2D. We also controlled for potential confounders, including night sleep, depression, and cognitive impairment. However, there are several limitations to this study. Participants were recruited from a community health care center. Their average age was 67 years, and most of them were retired, limiting the representativeness of the sample. Study findings cannot be generalized to the younger population. Different associations have been reported between napping and cardiometabolic health in young and middle-aged adults vs. older adults (66). In addition, exclusion criteria and napping were captured by self-report, which could introduce recall bias. We used HbA1c as a measure of overall glycemic control during the past three months, which only provides a snapshot, precluding us from having a closer examination of how daytime napping may influence glucose metabolism. Lastly, causality cannot be determined due to the cross-sectional design.

### 4.1 Implications for future research and clinical practice

Despite the above limitations, this study has implications for future research and clinical practice. In the future, studies conducted in young and middle-aged adults are needed to confirm findings from this study as they may have different napping behaviors. Future research may consider using ecological momentary assessment for napping and glucose to provide a more complete picture (e.g., sleep dairy, actigraphy, and continuous glucose monitor). Longitudinal and interventional studies targeting napping are needed to shed lights on the causal relationship between napping and glycemic control as well as the underlying mechanism (e.g., inflammation). Meanwhile, in clinical practice, healthcare professionals may offer tips about napping, e.g., taking a nap less than an hour, taking a nap in the afternoon instead of in the morning, avoiding appetitive napping.

## 5 Conclusions

In summary, this study suggests that daytime napping, when arranged inappropriately, might have detrimental effect on glycemic control among patients with T2D. Specifically, those with a long napping duration, morning napping timing, and appetitive napping may have a higher level of HbA1c. The present study provides new insights into glucose management and sleep-related intervention among people with diabetes. Meanwhile, further research is needed to establish the causal link between napping characteristics and glycemic control and investigate the potential underlying mechanisms.

## Data availability statement

The raw data supporting the conclusions of this article will be made available by the authors, without undue reservation.

## Ethics statement

The studies involving humans were approved by the Institutional Review Board of Shanghai Jiao Tong University School of Medicine (#SJUPN-201811). The studies were conducted in accordance with the local legislation and institutional requirements. The participants provided their written informed consent to participate in this study.

## Author contributions

JY: Data curation, Formal analysis, Investigation, Methodology, Resources, Software, Writing – original draft, Writing – review & editing. JW: Data curation, Formal analysis, Investigation, Methodology, Resources, Software, Writing – original draft, Writing – review & editing. YC: Investigation, Resources, Writing – original draft, Writing – review & editing. MZ: Investigation, Resources, Writing – original draft, Writing – review & editing. AZ: Investigation, Writing – original draft, Writing – review & editing. JD: Methodology, Writing – original draft, Writing – review & editing. JZ: Writing – original draft, Writing – review & editing. FL: Writing – original draft, Writing – review & editing. YW: Writing – original draft, Writing – review & editing. PC: Writing – original draft, Writing – review & editing. BZ: Conceptualization, Data curation, Formal analysis, Funding acquisition, Investigation, Methodology, Project administration, Resources, Software, Supervision, Validation, Visualization, Writing – original draft, Writing – review & editing.
