# Supplementary material for: The relationship between daytime napping and glycemic control in people with type 2 diabetes
Source: Front Endocrinol (Lausanne). 2024 Mar 5;15:1361906. doi: 10.3389/fendo.2024.1361906 (PMC10948433; doi:10.3389/fendo.2024.1361906)
Supplement: Supplementary file 1 [file Table_1.docx]

Supplementary Material

The relationship between daytime napping and glycemic control in people with type 2 diabetes

Jinjin Yuan†; Jinle Wang†; Yingdan Chen; Min Zhang; Aimei Zhao; Jing Du; Jiahui Zhang; Fan Liu; Yueying Wang; Pei Chen; Bingqian Zhu^*^

*** Correspondence:**

Bingqian Zhu, PhD, Research Associate Professor

School of Nursing, Shanghai Jiao Tong University

227 S Chongqing Rd, Shanghai, China, 200025

Email: [zhubq@shsmu.edu.cn](mailto:zhubq@shsmu.edu.cn)

Phone: 86-021-63846590

Orcid ID: 0000-0003-3997-7882

**Supplementary Table 1** Bivariate analyses examining relationship between napping characteristics and HbA1c level (N=226)

| **Variables** | **HbA1c (%)** | **p** |
| --- | --- | --- |
|  | **median (IQR)** |  |
| Napping frequency  None  1-2 days/week  3-4 days/week  5-7 days/week | 6.9 (6.4-7.8)  6.3 (5.7-7.6)  7.1 (6.1-8.4)  6.8 (6.2-7.9) | 0.160 |
| Long napping duration **^a^**  Yes  No | 7.1 (6.1-9.2)  6.6 (6.0-7.6) | **0.027** |
| Timing of napping **^b^**  morning (before 12 pm)  afternoon (12 pm to 4:30 pm) | 7.1 (6.6-10.0)  6.7 (6.0-7.8) | **0.031** |
| Type of napping **^a^**  Appetitive napping  Restorative napping | 6.3 (5.7-7.6)  6.9 (6.2-8.1) | **0.013** |

***Notes.*** ^a^ N=180, ^b^ N=177, participants with evening napping were excluded from the bivariate analyses as there were only three; IQR, interquartile range; Mann-Whitney U test and Kruskal-Wallis H tests were used.
